# Evaluating witness testimony: Juror knowledge, false memory, and the utility of evidence-based directions

**DOI:** 10.1177/13657127211031018

**Published:** 2021-09-16

**Authors:** Rebecca K. Helm

**Affiliations:** 3286 University of Exeter, Exeter, UK

**Keywords:** criminal procedure, legal directions, eyewitness memory, psychology and law, false memory.

## Abstract

Eyewitness evidence is often important in criminal cases, but false or misleading eyewitness evidence is known to be a leading cause of wrongful convictions. One explanation for mistakes that jurors are making when evaluating eyewitness evidence is their lack of accurate knowledge relating to false memory. This article examines lay beliefs relating to memory and ways in which they diverge from expert consensus. It identifies ways in which current directions provided to jurors in this area are likely to be deficient in influencing juror knowledge and in helping them apply that knowledge in a case context, and develops criteria that can be used to assess the likely effectiveness of directions. A new evidence-based training direction is designed based on these criteria, and tested in a mock jury study (N = 411). Results suggest that the proposed direction is more effective than a basic direction in influencing juror knowledge and facilitating the application of that knowledge to case facts.

Many modern common-law jurisdictions rely on lay juries to make determinations of fact in criminal cases (Helm and Hans, 2019). In making these determinations jurors are frequently required to hear and evaluate evidence from eyewitnesses to alleged crimes. Research suggests that jurors tend to rely heavily on this evidence (Penrod and Cutler, 1999). Traditionally, juries have been left to make judgments about the credibility of such evidence (including judgments of memory accuracy) without assistance from experts. In one House of Lord's judgment in England and Wales, Lord Hobhouse stated: ‘The courts should be cautious about admitting evidence from psychologists, however eminent, as to the credibility of witnesses. The assessment of the truth of verbal evidence, save in a very small number of exceptional circumstances, is a matter for the jury’ (*R v Pendleton*, 2002). This approach is designed to protect the independence of the jury and to avoid juror judgment being subsumed by expert judgment. It is based on the assumption that jurors, as people themselves, have experience that makes them well-placed to assess the credibility of other people (e.g. see *JH and TG*, ‘A witness’s ability to remember events, absent special considerations arising from the period of early childhood amnesia, will ordinarily be well within the experience of jurors.’)

However, research in both law and cognitive science shows that while laypeople do have their own experiences of memory, they face predictable difficulties when evaluating the memory of others. These difficulties are borne out in real cases—in both the United States and United Kingdom, for example, false or misleading eyewitness testimony has been identified as a leading cause of wrongful conviction (National Registry of Exonerations, 2021; UK Miscarriages of Justice Registry, 2021, see also Garrett, 2012; Wells et al., 2006). In many ways, these difficulties are not surprising. Years of research on memory in the behavioural sciences has shown that memory is highly complex, and that accurately distinguishing true and false memory (i.e. true memory from apparent recollection of something that did not actually occur) is hugely challenging. In fact, even experts in controlled experiments struggle to make such distinctions accurately (see, e.g., Ceci et al., 1994). The extent to which laypeople have experienced, and are conscious of having experienced, false memory is also unclear. False memory is not necessarily well within the experience of jurors.

Importantly for the jury system, research specifically shows that laypeople hold beliefs about false memory that are out of line with established and extensive empirical research that has been conducted in the area. As a result, jurors are likely to be examining memory with a poor understanding of the cues that suggest a memory could be false. Thus, jurors are likely to be making decisions that are clearly at odds with conclusions that would be drawn on the basis of established research. For example, in the case of *R v Hallam*, the appeals court noted that the eyewitness evidence upon which the jury had relied in convicting the defendant ‘was never very satisfactory’ (*R v Hallam*, 2012, para. 76). The fact jurors are provided with relatively little guidance when assessing memory makes it unsurprising that they make such mistakes, particularly when intuitive conceptions conflict with established knowledge in cognitive science. Presenting relevant information to jurors is likely to be helpful in this regard, but care must be taken to ensure that such information is presented in a way that (1) appropriately influences juror knowledge, and (2) can helpfully inform (but not dictate) their applied judgments in the context of a legal case. This paper focuses specifically on how legal procedure can facilitate the presentation of information in this way, specifically in cases involving potential false memory.

The first section of the paper examines likely discrepancies between layperson beliefs and empirical findings in the area of false memory in witnesses. The second part of the paper considers the extent to which current procedure relating to testimony addresses these discrepancies, with a focus on procedure in England and Wales. It suggests that to be effective in improving evaluations a direction must appropriately influence juror knowledge and facilitate the application of that knowledge in a case context. It then draws on psychological theory relating to memory and decision-making to show that current directions may be ineffective in both of these regards and to develop criteria for effective directions. The final part of the paper draws on these criteria to design and test an enhanced ‘training’ direction, compared to no direction or a basic direction, in a mock jury experiment. The results suggest that giving jurors a more detailed and evidence-based direction has the potential to minimise predictable weaknesses in their decision-making, without substituting trial by jury for trial by expert.

## Memory: Science versus public perception

Research over the last 40 to 50 years has provided extensive insight into the malleability of memory, showing that memory is reconstructive. What is remembered is influenced by a variety of factors including perception, imagination, semantic memory, and beliefs (e.g. Brainerd and Reyna, 2005; Howe and Knott, 2015; Loftus, 2003). Some of the ways that memory might be influenced are relatively intuitive and thus within the experience of jurors, while others may be less intuitive and therefore difficult to account for without additional guidance. Where jurors rely on their ‘common sense’ without knowledge of the less intuitive causes and characteristics of false memory, they are susceptible to making decisions based on presumptions that are demonstrably false and do not align with established scientific findings (see, e.g., Houston et al., 2013). Identifying areas in which lay opinion is likely to diverge from scientific consensus is important in understanding where jurors might need additional guidance in examining witness memory.

### The science of false memory

When a person witnesses an event, that event is encoded in memory. However, research clearly shows that memory does not operate like a video recorder and that a witness's memory will often not be a full and accurate description of an event. The accuracy of memory can be influenced by factors that affect how they encode what they have seen. These may include individual factors (such as whether the witness was scared or intoxicated) (e.g. Jores et al., 2019), circumstantial factors (such as how far away the event occurred from the witness and how well-lit the scene was) (Hope and Gabbert, 2019), and social factors (such as the sex, age group, and race of the witness and those involved in the event they are witnessing) (Yaros et al., 2019). These factors generally, with the potential exception of social factors, may be considered within the experience of laypeople. For example, people know that their memory for an event is likely to be worse when they viewed the event while intoxicated or stressed, while the lighting was bad, or at a significant distance.

People are less likely to have experienced, or at least to have been conscious of experiencing, corruption of their memory after encoding. However, research clearly shows that memories are not unchanging records of an event, that they can be altered once encoded, or even entirely implanted for events that did not take place (Clifasefi et al., 2007).

Memory can be influenced, sometimes referred to as corrupted, in a number of ways. First, by suggestion from an external influence. This external influence may be leading questions in an interview, (Loftus et al., 1978), conversations with others (Gabbert et al., 2003), or relevant media coverage (Davis and Loftus, 2012). A long line of research has even shown that encouraging a people to think about non-events (events that did not really happen) can eventually lead them to believe the events did happen (sometimes at relatively high rates) (see, e.g., Heaps and Nash, 2001; Thomas and Loftus, 2002; Wade et al., 2002). In some cases, the memories people report after having been influenced by suggestion are rich and detailed, and those reporting them have high confidence in their veracity (Loftus, 2003).

An important phenomenon thought to contribute to these memories is *source misattribution*. Source misattribution occurs where a person is unable to accurately separate two or more sources of their memories (Ceci et al., 1994). Thus, a person may come to believe something due to having considered or thought about it. People can come to believe that an event that they have imagined or thought about actually took place, or that a person they considered having been at a crime scene was definitely there. Essentially, the image that people create in their minds when thinking about the event or person is later confused for something that happened in reality.

Research also shows that false memories can arise more spontaneously. For example, a false memory can arise where a person remembers the ‘gist’ of something that they have seen (e.g. an offender) (creating a familiarity) but not specific ‘verbatim’ details (precise recollection) (Brainerd and Reyna, 2005). When this happens, a person can accept that a face they are shown is that of the offender where the face matches the relevant gist and so feels familiar, but sufficient verbatim details are not recalled to show that the familiar face is not a true match. This occurrence is not infrequent since gist memory for an event is thought to endure for longer than verbatim memory (Brainerd and Reyna, 2005). In some cases, a mistaken memory for verbatim details combined with familiarity (e.g., the familiarity of a face) can result in strong but false recollective experiences. This phenomenon is predicted by a psychological theory of memory and decision-making known as Fuzzy-Trace Theory (FTT), and has been termed ‘phantom recollection’ (Brainerd et al., 2001). It has the potential to result in the confident identification of a suspect who is ‘gist-consistent’ with the true offender, despite that person not actually being the offender.

Spontaneous false memory can also arise as a result of source monitoring errors (discussed above) or as a result of what is known as change blindness where a witness does not notice that perpetrator and a bystander are actually different people (Davis et al., 2008; Laney and Loftus, 2010). These errors can lead to an occurrence known as unconscious transference, or the familiar bystander effect. This effect refers to a memory error whereby a witness identifies a familiar, but innocent person, as an offender. For example, research has shown a tendency to misidentify an innocent bystander to a crime as an offender (see, e.g., Davis et al., 2008; Ross et al., 1994, 2006) or to identify a familiar person from an entirely different context as an offender (Thompson, 1988).

Scientific research therefore establishes that false memory is a real possibility even where memory encoding took place in pristine conditions, and even in the absence of problematic identification procedures. Memory can be influenced by information obtained after an event as well as the event itself, eyewitnesses sometimes identify as a culprit someone who they have seen in another situation or context, and confidence can be influenced by factors other than accuracy. Research conducted with memory experts has tested their endorsement of these, and other, propositions relating to memory. This research has generally shown memory experts to be supportive of these conclusions. For example, in one study conducted in 2006, 94% of the 64 memory experts surveyed agreed that memory reflects not only what a witness saw but also information obtained later on; 81% agreed that eyewitnesses sometimes identify as a culprit someone who they have seen in another situation or context, and 95% agreed that confidence could be influenced by factors other than memory accuracy (Benton et al., 2006, see also Kassin et al., 2001). However, although laypeople may show relatively high rates of agreement with experts when considering factors influencing memory encoding and problematic identification procedures (e.g. Desmarais and Read, 2011), their beliefs relating to false memory created in other ways are far less consistent with expert opinion.

### Public beliefs relating to false memory

Data gathered from surveys with laypeople suggests that their opinions diverge from those of memory experts when considering false memory (see, e.g., Kassin et al., 2001; Shaw et al., 1999; Simons and Chabris, 2012). In the 2006 study by Benton et al., referenced above, the views of laypeople differed from those of experts. Specifically, 60% of the 111 laypeople surveyed agreed that memory reflects not only what a witness saw but also information obtained later on (compared to 94% of experts); 30% agreed that eyewitnesses sometimes identify as a culprit someone who they have seen in another situation or context (compared to 81% of experts), and 50% agreed that confidence could be influenced by factors other than memory accuracy (compared to 95% of experts). Other research suggests that lay beliefs may come closer to expert beliefs, but still diverge relatively significantly. A 2012 meta-analysis examined responses from 23 surveys across multiple jurisdictions assessing lay knowledge of eyewitness issues that in total examined the beliefs of 4669 respondents (Desmarais and Read, 2011). Of the 4669 responses examined across multiple studies, 72% agreed that memory reflects not only what a witness saw but also information obtained later on; 63% agreed that eyewitnesses sometimes identify as a culprit someone who they have seen in another situation or context and 73% agreed that confidence could be influenced by factors other than memory accuracy. The meta-analysis concluded that layperson knowledge relating to memory was improving over time, but that important divergences still exist, particularly for estimator variables (variables not under the control of the legal system), evaluation of which may still be ‘beyond the ken’ of potential jurors.

Research also suggests more generally that laypeople are over-confident in the accuracy of memory. In one telephone survey participated in by 1838 people in the USA, 37.1% of respondents mostly agreed or strongly agreed that ‘In my opinion, the testimony of one confident eyewitness should be enough evidence to convict a defendant of a crime’, 63% of respondents mostly agreed or strongly agreed that ‘Human memory works like a video camera, accurately recording the events we see and hear so that we can review and inspect them later’, and almost 48% of respondents mostly agreed or strongly agreed that ‘Once you have experienced an event and formed a memory of it, that memory does not change’ (Simons and Chabris, 2011). None of the 73 experts surveyed agreed with any of these statements (Simons and Chabris, 2011). The statements are also inconsistent with evaluations of the courts themselves. For example, in the USA in the 2011 case of *Jersey v Henderson,* the court stated that ‘We are convinced from the scientific evidence in the record that memory is malleable, and that an array of variables can affect and dilute memory and lead to misidentifications.’ Therefore, the phenomenon of false memory is widely understood and accepted in the legal context, but not by those who are actually assessing the memory and credibility of witnesses. It is important to consider the extent to which this lack of understanding is accounted for and corrected by legal procedure.

## Eyewitness evaluation in England and Wales

### Current procedure

In 1976, a departmental committee chaired by Lord Devlin concluded that there was a special risk of wrongful conviction in cases reliant on eyewitness identification evidence due to the fact that even a witness who is sincerely convinced that they are making a correct identification may ‘not infrequently’ be mistaken (Devlin, 1976). Since that report was published, the courts and legislature in England and Wales have taken steps designed to reduce the risk of wrongful convictions arising as a result of mistaken identifications. In the area of juror evaluations, a specially constituted Court of Appeal issued guidelines for use in cases reliant on witness identification evidence (*R v Turnbull*, 1977). The guidelines oblige the court to halt cases in which the prosecution relies on very weak witness identification evidence, and to deliver a direction to the jury in other cases reliant entirely or substantially on witness identification (Bromby et al., 2007). This direction is usually delivered after witness testimony is presented to the jury, although an early or summary direction may be given at the outset of a case where helpful (Judicial College, 2020). The direction warns jurors of the special need for caution before convicting a defendant in reliance on the correctness of the identification. The direction can debunk relevant memory ‘myths’ (such as the fact that a confident witness is always reliable), list potential influential encoding factors, or explain relevant discrepancies in witness statements. The specific content of a Turnbull direction will vary by case. Details of how Turnbull directions should be given are contained in the Judicial College Crown Court Compendium (Judicial College, 2020). An illustrative example of Turnbull direction content is also given in this compendium, and is reproduced in Appendix 1.

In addition to the use of directions, it is possible to call expert witnesses to explain memory to jurors and judges. However, this is only permitted in rare cases, since it was held *in R v Turner* that where triers of fact can form their own opinion without the assistance of an expert, the matter in question being within their own experience and knowledge, opinion evidence of an expert is unnecessary and therefore inadmissible (*R v Turner*, 1975). Memory is generally considered to be within the experience of jurors (see *JH and TG*) and thus experts cannot be called to give an opinion on the likely deterioration of the memory of an ordinary witness (*R v Browning*, 1995). Thus, cases involving memory (with the exception of cases involving childhood amnesia, see *R v H (JR)* (2006)), are considered better dealt with by an educative judicial direction, as discussed above. However, existing research in the US context shows that judicial directions are not necessarily effective in achieving desired effects (Dillon et al., 2017; Papailiou et al., 2015). It is therefore important to carefully design and evaluate relevant directions.

### Evaluating current procedure

In order to be effective, directions relating to witness memory must both appropriately alter juror knowledge relating to witness memory, and facilitate the application of that knowledge in a case context. It is not clear that directions such as those mandated by *R v Turnbull* do either, and an examination of psychological theory relating to memory and decision-making suggests that they risk being ineffective. The continued persistence of miscarriages of justice since 1976, for example the cases of Joseph Otoo (Innocent.org, 2000), Victor Nealon (*R v Nealon*, 2014), John Kamara (*R v Kamara*, 2000), and Sam Hallam (*R v Hallam*, 2012), supports this contention (for more information on miscarriages of justice arising as the result of eyewitness identifications see Helm, 2021).

First, it is worth noting briefly that one problem with Turnbull guidelines is that their effectiveness depends heavily on the discretion of judges, who are themselves not memory experts. Research with judges in the United States (whose knowledge may differ from judges in England and Wales) highlights a risk that judicial knowledge may differ from expert knowledge in important ways (e.g. Wise and Safer, 2004). Difficulties are also evident in real cases, for example the case of *R v Hallam*, where a case was allowed to continue despite relying substantially on eyewitness evidence that the Court of Appeal noted was ‘never very satisfactory’. Increasingly, judges are being offered training designed to improve their ability to assess the potential for false memory and thus their ability to appropriately handle cases involving such memory. This ability might be further improved by updating the content of Turnbull directions to appropriately reflect more modern knowledge and circumstances (e.g. Ruva et al., 2007), and as a result allowing judges to deliver the most appropriate and up to date information to jurors. However, even where accurate information is presented to jurors in appropriate cases, there remain problems with delivering the information in the current form, as a relatively superficial judicial direction usually following the presentation of evidence.

#### Directions may not effectively influence juror knowledge

Existing directions are unlikely to effectively update juror beliefs relating to memory. In these directions, the conclusions of relevant research are presented as facts without sufficient underlying background to persuade jurors of their veracity and importance. In order for jurors to update their beliefs, it is likely that they will need more explanation and information, particularly since jurors do have their own ‘common-sense’ intuitions about memory that need to be dispelled. Psychological theory and research provide support for this contention. One relevant psychological model is Petty and Cacioppo's Elaboration Likelihood Model (ELM) (Cacioppo and Petty, 1980). This model is a dual process model describing attitude change. According to the model, attitude change can occur either via a central route or a peripheral route. Under the central route, attitude change results from a person's careful and thoughtful consideration of the merits of information. The central route involves an individual deeply considering and evaluating the information that they have received and coming to agree with it. Under the peripheral route, attitude change results from an association of the information with positive or negative cues that allow a person to make inferences about the merits of a position (e.g. assessments of the credibility of the source of information). The information currently presented in judicial directions such as Turnbull directions is not sufficiently detailed in terms of background and context to allow jurors to consider deeply for themselves whether they agree with the information in the directions, and therefore will not change juror beliefs via the central route. For example, telling jurors that a confident witness may be accurate does not give them meaningful information to enable them to evaluate and substantiate this claim. Thus, whether jurors update their beliefs based on a direction will depend on whether their attitudes are changed via the peripheral route. Whether attitude change occurs will therefore depend on the presence of cues that jurors feel comfortable drawing influences from. The legal system relies on jurors being sufficiently influenced by the ‘cue’ of judicial authority, and changing their beliefs based on the authority of the judge providing them with information. However, this cue may not be sufficient in the context of a legal case, where positions towards the case and the defendant may be polarised even prior to the directions being given. Research specifically suggests that beliefs in the authority and credibility of experts are influenced by existing beliefs (e.g. Kahan et al., 2011) and that initial impressions of a case can influence interpretations of information given subsequently (Carlson and Russo, 2001). There is therefore a significant risk that jurors will find the authority of the judge sufficiently important to update their beliefs only where doing so supports their existing evaluations of case evidence (which may also be influenced by biases and pre-existing attitudes, see e.g. Lecci and Myers, 2008), but not where it goes against them.

#### Directions may not effectively facilitate the application of information given to case facts

According to the Story Model, the leading model of jury decision-making, jurors decide cases through imposing a narrative ‘story’ organisation on trial information (Pennington and Hastie, 1981, 1986, 1988). Individual pieces of evidence are given meaning through their incorporation into narratives that explain what happened, and verdicts are reached through matching the best fitting narrative (measured in terms of coverage, coherence, uniqueness, and goodness-of-fit) with a verdict category (Pennington and Hastie, 1992). The juror decision-making process therefore involves fitting evidence into narratives, learning about verdict categories, and matching the story to the best fitting verdict category. Empirical research provides support for this explanation of decision-making processes in jurors. For example, research suggests that juror mental representations of evidence have a story structure (in which causal and intentional relations between different alleged events are central), that jurors are more likely to remember evidence that is consistent with the story associated with their verdict, and that jurors are likely to find evidence more important where that evidence has a causal role in the story that is associated with their verdict (Pennington and Hastie, 1992). This understanding of jury decision-making, combined with other research, highlights several deficiencies in existing directions and suggests that existing directions are unlikely to effectively facilitate the application of information given to case facts.

First, the information given in existing directions is not sufficiently detailed to allow jurors to get a good sense of when a particular concern might apply and how that concern might fit into an overall narrative. For example, jurors may be told that a confident witness is not necessarily accurate and that they should bear this in mind when considering the testimony of a particular confident witness. However, jurors are not given more detailed information about the types of situation in which existing research has shown that such witnesses may not be reliable, or to help them know how to evaluate whether a particular confident witness is accurate. This information is important, since the relationship between accuracy and confidence is complicated and is moderated by other factors (see e.g. Wells et al., 2006).

Second, there is a risk that the information presented in judicial directions is presented in a way that is too cursory to be effectively integrated into juror story construction. According to FTT, decision-making in adults is primarily driven by the *gist*, or meaning attributed to information, rather than the verbatim information itself (Reyna, 2012). FTT extends the original story model by introducing the broader concept of mental representation. The gist of the narrative (the ‘story’) connects all of the dots together and is the highest level of gist However, gist is not just the end product of a deliberative process but is extracted throughout the process from individual pieces of evidence. Put simply, jurors are expected to rely on the meaning they extract from evidence rather than the verbatim evidence itself (e.g. Helm et al., 2017). Therefore, making evidence meaningful to jurors and ensuring the meaning that they extract from evidence is appropriate is key to facilitating informed decision-making. The importance of making directions meaningful to jurors is also confirmed by research from other contexts showing that jurors attend more to jury directions where it is explained *why* they are being given (Kassin and Sommers, 1997; Steblay et al., 2006).

However, current Turnbull directions do not present information to jurors in a way that is conducive to them extracting meaning from it appropriately. Conclusions from a complex body of research are presented in quite a simplistic way, as stated conclusions. Relatively limited information about the types of cues that may be indicative of whether a particular concern should apply in a particular case is given. For example, as noted above, a judge may warn the jury that even a witness who is very confident may be incorrect. However, without being told why this might be the case or being given examples of the types of other case in which memory has been impaired in this way, it is likely to be difficult for jurors to extract meaning from it appropriately. This lack of contextualisation could lead to (1) jurors either being dismissive of all witness evidence (an effect that has been demonstrated when examining instructions on eyewitness testimony in the US context, see e.g. Dillon et al., 2017; Papailiou et al., 2015), (2) jurors not attributing meaning to the information at all and therefore neglecting it in their decision making, or (3) jurors attributing meaning to the information in a biased way—judging the importance of the warning based on their unrelated conceptions of the case (e.g. if they feel overall that the defendant seems guilty they might conclude that although confident witnesses can be wrong, the confidence in the case at hand does signal accuracy). This phenomenon can be caused by motivated cognition (see e.g. Kahan et al., 2011) and confirmatory biases (Carlson and Russo, 2001).

Third, where directions are not given prior to the presentation of evidence, they risk being ineffective. Where a judge gives the jury instructions on matters of law, for example on the presumption of innocence and the standard of proof, this information forms part of the story classification stage where jurors match their story with a verdict category (Pennington and Hastie, 1992). So, a story will be a match for the guilty verdict category only if that story is considered a fit ‘beyond a reasonable doubt’. However, where a judge gives information relating to the quality of evidence in a case, this information relates to the story construction and therefore needs to inform story construction in order to be effective. However, it is difficult for the information to influence story construction when it is presented after story construction has taken place for individual jurors. Presenting the information after story construction presents a risk that the value attributed to information will be coloured by its fit with the narratives that jurors have already begun constructing. The suggestion that directions should consistently be given at the outset of a case in order to ensure effectiveness is supported by an existing body of research showing that directions are more effective when given ‘pre-instruction’, meaning before evidence is presented (Chalmers and Leverick, 2018).

### Criteria for more effective directions

If the contentions described above are correct, directions given to jurors should be more effective in updating beliefs and facilitating appropriate application to facts if they:

- Provide jurors with sufficient information to allow them to evaluate the case for the conclusions presented and update their beliefs accordingly;
- provide jurors with sufficient information to allow them to understand the types of case in which particular concerns may be important, and why; and
- are consistently given prior to the presentation or case evidence in order to inform juror narrative construction from evidence, and to reduce effects of motivated cognition or confirmatory bias in juror interpretation and analysis of information given.

Essentially, directions should be viewed as a form of training for jurors rather than relatively simple warnings. This training has the potential to empower jurors with the information and context needed to more appropriately assess testimony in a case context. The suggestion of training jurors has already been made in other contexts (see, e.g., Koehler, 2006; Reed, 2007).

In the context of complex numerical intervention, suggestions to help jurors truly understand the meaning of information they are presented with, have included using visual aids and graphs (Helm et al., 2017; see also Gaissmaier et al., 2012). These visual aids allow jurors to more appropriately grasp bottom-line meaning of information. In the context of eyewitness testimony, such aids are unlikely to be appropriate since the information cannot be communicated graphically. Instead, conveying key information on the research behind directions relating to memory and clear representative examples of cases in which memory errors have been found should be considered. For example, rather than telling jurors that it is possible for a witness to mistakenly identify a defendant because of having seen or heard about the defendant in another context, a judge might tell jurors that memory mistakes occur where a person mistakenly identifies someone who feels familiar to them, this effect occurs because of what are known as source monitoring mistakes…, these are the kind of cases that the effect has been demonstrated in… In this way, the information relating to memory becomes a meaningful tool that jurors can use to appropriately inform their evaluation of evidence as it is presented. This type of direction will be referred to as a ‘training direction’.

The next section of this paper tests a short training direction and analyses its influence on decisions when compared to no direction being given, and when compared to a briefer direction more like a Turnbull direction, but presented prior to case evidence.

## Testing evidence-based directions: A mock-jury experiment

### Methods

#### Research design

An online mock jury experiment utilising a between-subjects design was conducted in order to test the potential effectiveness of an enhanced training direction on juror decisions. Mock jurors each read a brief case vignette in a case reliant almost exclusively on evidence from two witnesses. The experiment utilised a 2 (witness evidence strength) × 3 (direction given) design. Vignettes participants read varied in terms of the strength of the witness evidence in the case (strong eyewitness evidence or weak eyewitness evidence), and the direction jurors received (no direction, a basic direction, or a ‘training’ direction designed in accordance with the criteria above). Mock jurors delivered a verdict in the case, and rated their agreement with statements about eyewitness memory (which was examined to assess how directions influenced juror knowledge).

#### Materials

*Directions*. The basic directions were designed to present mock jurors with research conclusions from a judge, without giving background information or examples. Jurors were told that they should consider the fact that in cases involving eyewitness evidence there was a risk of injustice and so care should be taken when considering the evidence. They were also given a number of more specific warnings—that a witness who is honest and convinced of something may still be wrong, that a convincing witness may still be wrong, that a witness who recognises a defendant may be wrong even where they know the defendant well, and that it is possible to mistakenly identify a defendant because of having seen or heard about them in another context. The training directions focused on specifically describing research on memory to jurors, in an attempt to illustrate the same information about memory but in a more contextualised way. Jurors were told that research on memory distortion has shown that information obtained after an event can change what a person remembers and even create false memories, and that memory can be inaccurate even when a witness is being honest. They were then given accessible examples from research on false memories and descriptions of explanations given for these false memories having been formed (specifically focused on source monitoring and change blindness, described above). Full versions of both directions are included in Appendix 2.

*Case vignettes.* The case vignettes utilised were loosely based on the case of Sam Hallam, who was convicted of murder in 2005 largely on the basis of witness testimony. This conviction was quashed on a second appeal in 2012 (see *R v Hallam*, 2012). The vignettes used were simplified and involved only two of the four witnesses at the original trial (the two witnesses identified by the Court of Appeal as being the key witnesses in the case). This simplification was done in order to minimise confounding by other case features, so that analyses could focus closely on specific aspects of witness testimony. The case was therefore used not because this work was intended to be an accurate replica of it or to lead to conclusions on it, but just because it provided a realistic case setting within which to explore potential eyewitness issues. In the case, a young person was killed in a fight involving a large number of youths. The defendant was linked to the attack by evidence from two eyewitnesses, and had initially given an incorrect alibi.

In the strong witness evidence condition, both witnesses linked the same person (the defendant) to the murder immediately after the event (one having seen him hit the witness with the murder weapon, and another having seen him moving towards the victim with the murder weapon immediately before the attack) and without colluding with one another. There were no indications given of any external influence with the potential to corrupt memory. Therefore, in this case a greater understanding of false memory would not necessarily be expected to result in a reduction in guilty verdicts.

In the weak witness evidence condition, the first witness did not mention the defendant initially and only identified him after she had seen him in the street the next day and the second witness did not mention the defendant initially and only identified the defendant after he had heard his name from the first witness. The weak evidence condition was designed to represent a situation with a clear possibility of memory corruption following the event itself, based on the research discussed above. To ensure this case did represent a case in which the eyewitness evidence, in the opinion of memory experts, would not substantiate a guilty verdict, an initial pilot of the case materials was run with memory experts. Eight memory experts reviewed the case, having been recruited via emails sent to academics working on research relating to memory and law at PhD level or above (identified by being authors of academic publications in memory and law, or being researchers in the research groups of authors of academic publications in memory and law). When asked to indicate whether they thought that the defendant was guilty beyond reasonable doubt, all of the memory experts indicated that they thought that the defendant was not guilty.

*Memory questions.* Participants were asked to rate their agreement with statements relating to false memory examined in eyewitness research discussed in Part 1 of this paper. Specifically, participants were asked to rate their agreement (agree, disagree, don't know) with the following statements:

- Eyewitness testimony about an event often reflects not only what they actually saw but also information they obtained later on.
- Eyewitnesses sometimes identify as a culprit someone they have seen in another situation or context.
- An eyewitness’s confidence can be influenced by factors that are unrelated to identification accuracy.

In order to ensure that expert consensus continues to suggest that each of these statements are true, we asked each of the eight memory experts who reviewed our case materials to indicate their agreement with the statements. 87.5% of the experts (7/8) agreed with the first and third statements, and all of the experts agreed with the second statement.

#### Participants

Participants were 411 adults, recruited online using the Prolific survey platform. Participants completed the study online, and participation took approximately 15 minutes. Participants ranged in age from 18 to 86 (*M* = 35.13, *SD* = 12.87). All participants were British. 89.8% identified as White, 3.9% identified as Asian, 2.9% identified as Black, and 3.4% identified as another ethnic group or preferred not to indicate their ethnic group. 27.3% identified as male, 72.3% identified as female, and 0.5% identified as another gender group (e.g. non-binary).

#### Procedure

Participants were first shown their instruction (if applicable) (no instruction, basic instruction delivered by judge, or training instruction delivered by judge) followed by the case facts and evidence (either the weak case or the strong case). They were given an instruction on the relevant law (requiring the defendant to have killed another person with the intention to kill or the intention to cause grievous bodily harm on order to be guilty) and standard of proof (beyond reasonable doubt) and were asked to indicate whether they would find the defendant guilty or not guilty. They were also asked to indicate whether they thought that the defendant was one of the people responsible for the murder (definitely yes, probably yes, might or might not, probably not, or definitely not). After delivering a verdict they were asked some questions about their perceptions of the case evidence and were asked to rate their agreements with the memory statements.

#### Ethical considerations

This research gained ethical approval from the independent ethics committee at the University of Exeter. Participation was anonymous and all participants provided informed consent and were informed of their right to withdraw.

### Results

#### Do directions influence knowledge?

First, analyses examined differences in the agreement with the three memory statements presented to participants, in order to assess how the directions influenced juror knowledge relating to false memory. For each of the three memory statements, logistic regression analyses were used to examine the extent to which each direction increased agreement with the statement when compared to the no direction condition. The direction variable was dummy coded and entered into analysis predicting statement agreement as a no direction/basic direction comparison, and a no direction/training direction comparison. Analyses were also conducted controlling for evidence strength, and the significance of predictors was not affected.

*Eyewitness testimony about an event often reflects not only what they actually saw but also information they obtained later on.* 67.8% of participants in the no direction condition agreed that eyewitness testimony about an event often reflects not only what they actually saw but also information they obtained later on. Regression results did not indicate a greater proportion of participants endorsing this statement in the basic direction condition when compared to the no direction condition. Results did indicate a greater proportion of participants endorsing this statement in the training direction condition when compared to the no direction condition, although this effect just missed two-tailed statistical significance (*B* = .52, *SE* = .27, Wald = 3.74, *OR* = 1.69, *p* = .05). 78% of participants who received the training direction agreed with this statement compared to the 67.8% agreement rate in the no direction group (χ^2^ = 3.78, *p* = .05).

*Eyewitnesses sometimes identify as a culprit someone that have seen in another situation or context.* 48.6% of participants in the no direction condition agreed that eyewitnesses sometimes identify as a culprit someone that they have seen in another situation or context. Regression results did not indicate a greater proportion of participants endorsing this statement in the basic direction condition when compared to the no direction condition. However, results did indicate a greater proportion of participants endorsing this statement in the training direction condition when compared to the no direction condition (*B* = .95, *SE* = .25, Wald = 14.49, *OR* = 2.58, *p *< .001). 70.9% of participants who received the training direction agreed with this statement compared to the 48.6% agreement rate in the no direction group (χ^2^ = 14.80, *p *< .001).

*An eyewitness's confidence can be influenced by factors that are unrelated to identification accuracy.* 74.0% of participants in the no direction group agreed that an eyewitness's confidence can be influenced by factors than are unrelated to identification accuracy. Regression results did not indicate a greater proportion of participants endorsing this statement in the basic direction condition when compared to the no direction condition. However, results did indicate a greater proportion of participants endorsing this statement in the training direction condition when compared to the no direction condition (*B* = .82, *SE* = .31, Wald = 6.89, *OR* = 2.26, *p* = .01). 86.5% of participants who received the training direction agreed with this statement compared to the 74.0% agreement rate in the no direction group (χ^2^ = 7.10, *p* = .01).

**Figure 1.**
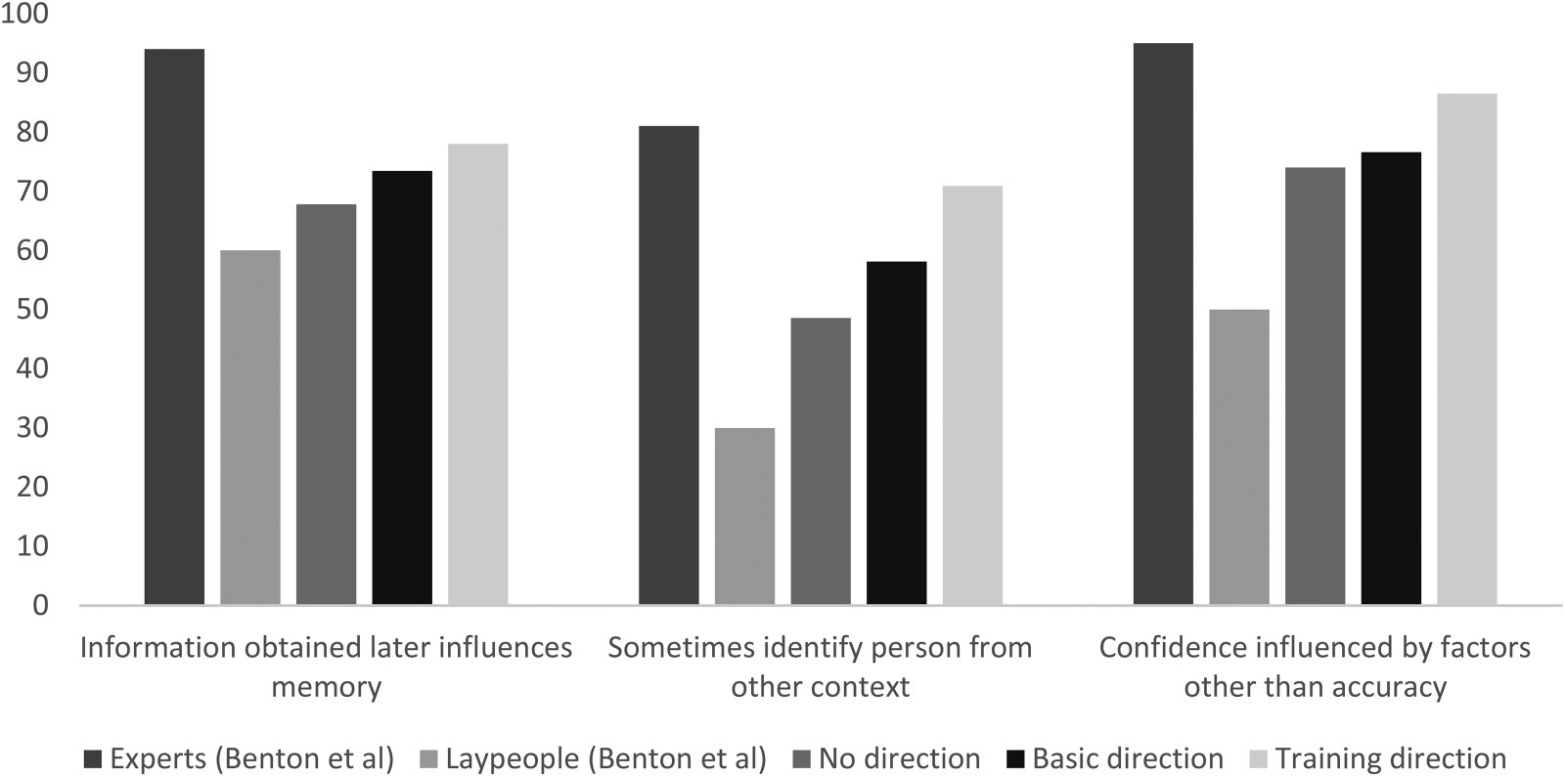
Agreement rates with statements about memory from this study, and Benton et al. (2006).

Figure 1 displays agreement rates with each of the three statements in each of the three direction conditions in this experiment alongside the expert and layperson agreement rates presented in Benton et al., 2006 (that utilised the same questions and response scale). Even participants in the no direction condition tended to agree with the statements more than those in the Benton et al., study. This difference could reflect increasing awareness of eyewitness memory inaccuracy in laypeople (see Desmarais and Read, 2011).

Three follow-up logistic regression analyses confirmed that endorsement of each of the statements relating to eyewitness testimony was associated with verdict (regressions were run separately due to covariance between endorsements of each statement). In each regression, statement endorsement significantly predicted verdict such that endorsement was associated with a lower chance of delivering a guilty verdict (Eyewitness testimony about an event often reflects not only what they actually saw but also information they obtained later on: *B* = 1.06, *SE* = .24, Wald = 20.20, *OR* = 2.88, *p *< .001; Eyewitnesses sometimes identify as a culprit someone that have seen in another situation or context: *B* = .78, *SE* = .21, Wald = 14.64, *OR* = 2.19, *p *< .001; An eyewitness's confidence can be influenced by factors that are unrelated to identification accuracy: *B* = 1.05, *SE* = .26, Wald = 16.39, *OR* = 2.87, *p *< .001).

#### Do directions influence application of knowledge (verdicts)?

A logistic regression analysis was conducted to examine the influence of direction received and evidence strength on verdicts. If the directions are effective at facilitating application of the information given, we would expect them to influence verdicts in the weak evidence condition (where the information contained provides reasons to question the witness evidence) but to have less of an influence or no influence on verdicts in the strong condition (where the information contained provides little or no reasons to question the witness evidence). Predictors were evidence strength (weak or strong), two direction dummy variables (as above, direction was dummy coded in analyses into two comparison variables—a no direction/basic direction comparison, and a no direction/training direction comparison), and the interaction between evidence strength and each of the direction dummy variables. The outcome variable was verdict (guilty or not guilty).

This analysis revealed a significant main effect of evidence strength such that the rate of participants delivering a guilty verdict was significantly higher in the strong evidence condition (*B* = −1.31, *SE* = .36, Wald = 13.14, *OR* = .27, *p *< .001). 73.4% of participants in the strong evidence condition found the defendant guilty compared to 30.8% of participants in the weak evidence condition (χ^2^ = 73.74, *p *< .001).

The analysis also revealed a main effect of the no direction/training direction comparison, such that participants who received the training direction were significantly less likely to find the defendant guilty than participants who received no direction (*B* = .83, *SE* = .34, Wald = 5.82, *OR* = 2.28, *p* = .016). 44.7% of participants in the training direction condition found the defendant guilty compared to 54.1% of participants in the no direction condition (although this difference was not significant in a simple chi-square analysis; χ^2^ = 2.55, *p* = .110).

**Figure 2.**
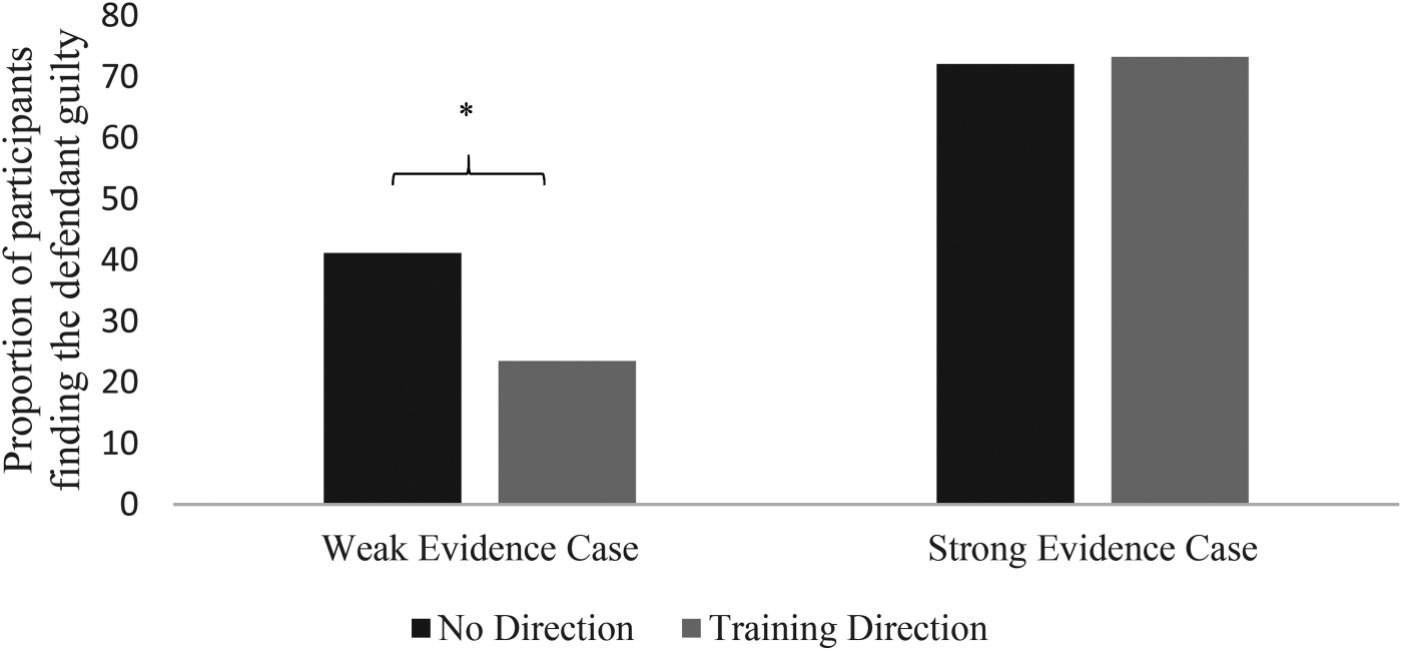
Guilty verdicts by evidence condition and training direction.

The analysis also revealed a two-way interaction between evidence strength and the no direction/training direction comparison, that just missed two-tailed significance (*B* = −.89, *SE* = .53, Wald = 2.77, *OR* = .41, *p* = .096) (Figure 2). This effect was explored further since it was a predicted result (since the training direction, if effective, should reduce guilty verdicts in the weak evidence condition but not in the strong evidence condition). Follow-up chi-square analyses showed that the training direction did significantly reduce the proportion of guilty verdicts in the weak evidence condition (41.2% of participants in the no direction condition found the defendant guilty compared to 23.5% of participants in the training direction condition; χ^2^ = 5.93, *p* = .01), but did not have an influence on the proportion of guilty verdicts in the strong evidence condition (72.1% of participants in the no direction condition found the defendant guilty compared to 73.3% of participants in the training direction condition; χ^2^ = .02, *p* = .88).

No other effects in the regression analysis were significant, indicating that the basic direction did not significantly change the rates of guilty verdicts. A follow-up linear regression analysis using the same predictors to predict judgments of how likely it was that the defendant was one of the youths responsible for the victim's death showed that both directions significantly decreased the extent to which mock jurors thought that the defendant was responsible (Basic Direction *B* = .28, *SE* = .13, *β* = .15, *t*(5405) = 2.15, *p* = .032; Training Direction *B* = .432, *SE* = .12, *β* = .25, *t*(5405) = 3.62, *p *< .001). This suggests that both suggestions may have some effect on beliefs relating to guilt, although only the training direction had a strong enough effect for this to translate into a change in verdict.

### Discussion

The results of this study provide support for the proposed use of more in-depth directions in relation to false memory in cases largely reliant on eyewitness testimony. The training direction tested here increased agreement with scientific consensus on memory in all of the statements examined (although note that this increase just missed statistical significance in the case of ‘Eyewitness testimony about an event often reflects not only what they actually saw but also information they obtained later on.’). The basic direction did not significantly increase agreement with scientific consensus, although qualitatively it did increase agreement, suggesting that such a direction may have a smaller effect but that this study did not have the power to detect it. In any case, the training direction was clearly more effective than the basic direction at increasing the extent to which juror beliefs were aligned with scientific consensus. Future research should fine-tune instructions to be maximally informative in relevant areas.

In addition, findings showed the training direction, but not the basic direction, influenced verdicts in the case. Specifically, in the weak evidence condition the training direction reduced the number of guilty verdicts, bringing the mock juror verdicts in line with expert judgment from the pilot study. Again, it may be that the basic direction did have a small effect that was not detected in this study, but the training direction clearly appears to be more effective in informing juror judgments. In the strong evidence condition, neither training direction significantly influenced the proportion of guilty verdicts. This finding suggests that the training direction is effective in helping jurors effectively identify an enhanced risk of false memory, rather than in just introducing general scepticism towards any eyewitness testimony.

The mock jury study presented here should be viewed in light of some limitations. Most importantly, the presentation of case evidence differed from presentation in real cases in a number of ways and thus the study was limited in external validity (Lieberman et al., 2016). First, the case facts reviewed by participants were presented in summary form and read by participants, rather than being presented through direct and cross examination in court. Second, the materials were significantly simplified compared to the materials that would be viewed in a full trial. Third, mock jurors were aware that their decisions would not have implications for real people. As a result, the actual rates of guilty verdicts delivered by jurors are likely to be different from the rates that might be expected if a similar case were heard in court. However, the experiment isolated and studied an important component of juror decision-making, the evaluations of testimony involving potential false memory. The same basic decision processes are likely to underlie this part of juror decision-making in real cases (e.g. Sklansky, 2013) and research suggests that effects found when evidence is presented in written format are likely to persist when evidence is presented in a more realistic format (McAuliff et al., 2009). Thus, the research is informative despite the limited external validity. It should also be noted that in real cases jurors deliver verdicts as a group following deliberation processes and this study did not incorporate deliberations. Future work should consider how the effects demonstrated might be influenced by deliberations, but research generally shows that individual juror verdicts are highly predictive of jury verdicts delivered following deliberations (e.g. Devine et al., 2016). This work therefore provides important initial insight that can be followed up by examining decisions in more realistic mock trials in future work.

Translating findings into real procedure would involve both altering the content of directions and the timing of delivery. All directions in this study were delivered to participants prior to viewing case evidence which differs from current practice. As noted above, theory and previous research confirm that instructions delivered prior to the evaluation of evidence are likely to be more effective (see Chalmers and Leverick, 2018). Therefore, the effectiveness of the directions tested may be dependent on directions being given prior to evidence being presented. Although this may mark a change from typical practice, existing Judicial College guidance does recognise the possibility of delivering an early direction (Judicial College, 2020). This recognition provides a basis to build on in making early delivery consistent practice.

## Conclusion

Years of well-controlled experimental research, as well as evidence from real miscarriages of justice, shows that memory is reconstructive, that witnesses are susceptible to memory corruption following an event, and that witnesses can come to have false memories relating to an event. This research is informative in the legal system, where evaluations of witness testimony are important in the adjudication of crime. However, care must be taken to incorporate this evidence into the legal system in a way that will ensure that it is effective in improving adjudication. In the case of juries, it cannot be presumed that giving jurors relatively brief warnings about witness testimony will appropriately inform their beliefs or influence their judgments. In fact, experimental research testing juror instructions in the United States (e.g. Dillon et al., 2017) and psychological theory relating to memory and decision-making both suggest that simple directions given to jurors by judges are unlikely to work. Instead, directions should be developed that contextualise work on memory, provide illustrative examples from research and explain phenomena that have been observed in a simplified way. Such directions give jurors a sense of the gist of false memory—what it can look like, why it occurs, and how we know it exists. In this way the directions are sufficiently informative to update juror beliefs, and arm jurors with tools and context they can use in evaluating memory. Where jurors have understandable and contextualised information to use in evaluations, they are more likely to be influenced by this information and less likely to be influenced by biases and misperceptions.

The directions explored in this study provide a starting point that can be built on in developing the directions provided to jurors both in England and Wales and in jury systems across the world. The direction here addressed potential false memory arising when there has been a delay between the observation of an event and the memory report. However, similar directions could be used in other areas in which juror judgments are likely to be influenced by misconceptions. For example, a common-sense approach suggests that witnesses who are inconsistent are likely to be lying or mistaken. However, research shows this inconsistency may be predictable in cases involving victims of trauma, particularly children, where memory blending can occur (Helm et al., 2018). More broadly, cohesive directions providing context and examples could even be helpful to dispel other ‘myths’ relied on by jurors, such as rape myths (e.g. Leverick, 2020).

Ultimately, more work is needed to translate these findings into appropriate policy for use in practice. Future research should consider how directions would be selected for inclusion in a particular case, or whether a generalised direction covering a range of information relating to memory could be effective in all cases largely reliant on memory. Directions should also be tested in a more realistic jury format, and across a wider range of cases. However, this paper makes the case that jurors are likely to be making decisions relating to memory that are at odds with established scientific research, that current directions are unlikely to be sufficient to correct this effectively, and that there are promising alternative and evidence-based solutions available.

## Appendix 1

### Turnbull direction example (Judicial College, 2020)

NOTE: Not all of the following directions need to be given in every case, as shown by the headings. It is suggested that the order is logical but it is for the judge to decide which directions are appropriate and the order in which they should be given.

In every case

You must be cautious when considering this evidence because experience has shown that any witness who has identified a person can be mistaken even when the witness is honest and sure that he/she is right. Such a witness may seem convincing but may be wrong.

[In a ‘recognition’ case: This is true even though a witness knows a person well and says that he/she has recognised that person. The witness could still be mistaken.]

You can only rely on the identification evidence if you are sure that it is accurate. You need to consider carefully all the circumstances in which D was identified. So you must ask yourselves:

- For how long could W see the person W says was D and, in particular, for how long could W see the person's face?
- How clear was W's view of the person, considering the distance between them, the light, any objects or people getting in the way and any distractions.
- Had W ever seen D before the incident? If so, how often and in what circumstances? If only once or occasionally, had W any special reason for remembering D?
- How long was it between the time of the incident and the time when W identified D to the police?
- Is there any significant difference between the description W gave of the person and D's appearance?

You should also think about whether there is any evidence which, if you accept it, might support the identification. In particular you should consider {specify}.

However, the evidence of {specify} cannot support the identification because {explain}.

You will also have to look to see if there are any weaknesses in any of the identification evidence, or if there is any evidence which, if you accept it, might undermine the identification evidence. In particular, you should consider {specify}.

### In a case where there has been evidence of identification and description

In this case you have identification evidence and description evidence.

Identification evidence is where a witness has identified a specific person by {for example naming the person/pointing the person out (whether in the street or at an identification procedure)}.

Description evidence is where a witness has given a description which may or may not be similar to the appearance or clothing of a particular person. However, the description alone does not identify that person, so it can only go to support other evidence, including evidence of identification.

### Where there has been an issue arising from a VIPER identification procedure

You have heard that D was picked out on a VIPER identification procedure from a number of images that had been selected by D and D's solicitor. {Summarise issue/s arising and evidence relating to those issues.}

### Where the defence is alibi

I have already explained how you should consider the evidence of D's alibi.

If you decide that D lied about where he/she was, this does not prove that W's identification must be right. But if you decide that D had no innocent reason for putting this alibi forward, you may treat D's false alibi as some support for W's identification.

Of course, if you are sure that W's evidence of identification is reliable, it would follow that D's alibi is false.

### Where there has been a breach of Code D

The fact that no identification procedure took place broke the rules that should be followed in cases involving disputed identification. These rules, known as the Code of Practice, are designed to provide safeguards for a suspect whom a witness says he/she can identify, and to test the ability of the witness to identify the suspect.

The failure to hold a formal identification procedure has deprived D of an important safeguard which would have tested W's ability to make an identification under formal and fair conditions. You must bear that in mind when considering the reliability of W's identification.

As no identification procedure was carried out in this case, W's ability to identify a suspect was not tested in this way and D has not had the advantage D might have had if W had failed to pick D out or had picked out another person.

You should take all this into account when you decide whether or not you can be sure that W's identification of D was reliable, and you should ask yourselves whether the fact that there was no formal identification procedure puts the identification evidence in doubt.

## Appendix 2

### Basic direction

Before you read the case facts, the judge gives this information:

Eyewitness evidence is important in this case. You should be aware that when considering cases involving eyewitness evidence there is a need for caution to avoid the risk of injustice.

- A witness who is honest and convinced in his or her own mind may be wrong.
- A witness who is convincing may be wrong.
- A witness who is able to recognise the defendant, even when the witness knows the defendant very well, may be wrong.

It is possible for a witness to mistakenly identify a defendant because of having seen or heard about the defendant in another context. You should consider this possibility when evaluating the case evidence.

### Training direction

Research on memory distortion has shown that information obtained after an event can change what a person remembers, and even create false memories. Anyone's memory for an event can be inaccurate, even when the person is honestly reporting what they think that they saw.

One type of memory inaccuracy occurs where a person mistakenly identifies someone as having done something because the person feels familiar to them. This can happen where the witness to an event sees a face that is familiar to them, and mistakenly links it to that event. In the context of witnesses to crimes, this means that people sometimes misidentify people who they saw at the scene of a crime or after a crime, as people who committed the crime.

Researchers have identified a number of reasons that this happens:

First, the effect can occur from what are known as source monitoring mistakes. Source monitoring mistakes happen where people accidentally link experiences with the wrong source, for example where they think something happened in real life when in actually happened in a dream. In the same way, when people see a person that is familiar, they can be mistaken about why the person is familiar. In criminal cases, this can result in a witness to a crime linking an innocent but familiar person with the crime. A sense of familiarity with the person leads the witness to identify them incorrectly.

For example, in one case, an eyewitness expert was accused of being the person who committed a crime, by a victim who had seen him on television. The television exposure of the expert made him familiar to the witness, but the witness confused the source of the familiarity.

Second, the effect can occur where during the encoding of memory a witness thinks that two people are actually the same person. For example, if a witness sees one person pick up a computer and walk out of a room and then sees another person walk back into the room with the computer, the witness may mistakenly believe that the people are the same person. In criminal cases, this can result in a witness to a crime mistakenly thinking that someone did a crime when actually they were only present at the scene of the crime.

For example, in one research study, people viewed a theft in a supermarket. The person who did the theft walked into a supermarket aisle and an innocent person then emerged from that aisle. More than half of the people who viewed the event mistakenly thought that the innocent person and the person who did the theft were the same person, and therefore mistakenly identified the innocent person as a criminal.

It is important to consider the possibility for misidentifications of this type when evaluating witness testimony.

## References

[bibr2-13657127211031018] Benton TR Ross DF Bradshaw E , et al. (2006) Eyewitness memory is still not common sense: Comparing jurors, judges and law enforcement to eyewitness experts. Applied Cognitive Psychology: The Official Journal of the Society for Applied Research in Memory and Cognition 20(1): 115–129. 10.1002/acp.1171

[bibr3-13657127211031018] Brainerd CJ Reyna VF (2005) The Science of False Memory. New York: Oxford University Press.

[bibr4-13657127211031018] Brainerd CJ Wright R Reyna VF , et al. (2001) Conjoint recognition and phantom recollection. Journal of Experimental Psychology: Learning, Memory, and Cognition 27(2): 307–327. 10.1037/0278-7393.27.2.307 11294434

[bibr5-13657127211031018] Bromby M MacMillan M McKellar P (2007) An examination of criminal jury directions in relation to eyewitness identification in commonwealth jurisdictions. Common Law World Review 36(4): 303–336. 10.1350/CLWR.2007.36.4.303

[bibr6-13657127211031018] Cacioppo JT Petty RE (1980) Persuasiveness of communications is affected by exposure frequency and message quality: A theoretical and empirical analysis of persisting attitude change. Current Issues and Research in Advertising 3(1): 97–122. 10.1080/01633392.1980.10505295

[bibr7-13657127211031018] Carlson KA Russo JE (2001) Biased interpretation of evidence by mock jurors. Journal of Experimental Psychology: Applied 7(2): 91–103. 10.1037/1076-898X.7.2.91 11477983

[bibr8-13657127211031018] Ceci SJ Loftus EF Leichtman MD , et al. (1994) The possible role of source misattributions in the creation of false beliefs among preschoolers. International Journal of Clinical and Experimental Hypnosis 42(4): 304–320. 10.1080/00207149408409361 7960288

[bibr9-13657127211031018] Chalmers J Leverick F (2018) Methods of Conveying Information to Jurors: An Evidence Review. Edinburgh: The Scottish Government. https://eprints.gla.ac.uk/161562/1/161562.pdf

[bibr10-13657127211031018] Clifasefi SL Garry M Loftus EF (2007) Setting the record (or video camera) straight on memory: The video camera model of memory and other memory myths. In: Della Sala S (ed) Tall Tales About the Mind and Brain: Separating Fact From Fiction. New York: Oxford University Press, pp.60–75.

[bibr11-13657127211031018] Davis D Loftus EF (2012) Inconsistencies between law and the limits of human cognition: The case of eyewitness identification. In: Nadel L Sinnott-Armstrong WP (eds) Oxford Series in Neuroscience, Law and Philosophy. Memory and Law. Oxford: Oxford University Press, pp.29–58.

[bibr12-13657127211031018] Davis D Loftus EF Vanous S , et al. (2008) ‘Unconscious transference’ can be an instance of ‘change blindness’. Applied Cognitive Psychology: The Official Journal of the Society for Applied Research in Memory and Cognition 22(5): 605–623. 10.1002/acp.1395

[bibr13-13657127211031018] Desmarais SL Read JD (2011) After 30 years, what do we know about what jurors know? A meta-analytic review of lay knowledge regarding eyewitness factors. Law and Human Behavior 35(3): 200–210. 10.1007/s10979-010-9232-6 20461542

[bibr14-13657127211031018] Devine DJ Krouse PC Cavanaugh CM , et al. (2016) Evidentiary, extraevidentiary, and deliberation process predictors of real jury verdicts. Law and Human Behavior 40(6): 670–682. 10.1037/lhb0000209 27598561

[bibr15-13657127211031018] Devlin LP (1976) Report to the Secretary of State for the Home Department on the Departmental Committee on Evidence of Identification in Criminal Cases. London: HMSO.

[bibr16-13657127211031018] Dillon MK Jones AM Bergold AN , et al. (2017) Henderson instructions: Do they enhance evidence evaluation? Journal of Forensic Psychology Research and Practice 17(1): 1–24. 10.1080/15228932.2017.1235964

[bibr17-13657127211031018] Evidence-Based Justice Lab (2021) UK Miscarriages of justice registry. Evidencebasedjustice.exeter.ac.uk. Available at: https://evidencebasedjustice.exeter.ac.uk/miscarriages-of-justice-registry/the-cases/overview-graph/ (accessed 13 April 2021).

[bibr18-13657127211031018] Gabbert F Memon A Allan K (2003) Memory conformity: Can eyewitnesses influence each other’s memories for an event? Applied Cognitive Psychology 17(5): 533–543. 10.1002/acp.885

[bibr19-13657127211031018] Gaissmaier W Wegwarth O Skopec D , et al. (2012) Numbers can be worth a thousand pictures: Individual differences in understanding graphical and numerical representations of health-related information. Health Psychology 31(3): 286–296. 10.1037/a0024850 21842998

[bibr20-13657127211031018] Garrett BL (2012) Convicting the Innocent: Where Criminal Prosecutions go Wrong. Cambridge, MA: Harvard University Press.

[bibr21-13657127211031018] Heaps CM Nash M (2001) Comparing recollective experience in true and false autobiographical memories. Journal of Experimental Psychology: Learning, Memory, and Cognition 27(4): 920–930. 10.1037/0278-7393.27.4.920 11486925

[bibr22-13657127211031018] Helm RK (2021) The anatomy of ‘factual error’ miscarriages of justice in England and Wales: A fifty-year review. Criminal Law Review 5: 351–373.

[bibr23-13657127211031018] Helm RK Hans VP (2019) Procedural roles: Professional judges, lay judges, and lay jurors. In: Brown D Turner J Weißer B (eds) The Oxford Handbook of Criminal Process. New York: Oxford University Press, pp.209–228.

[bibr24-13657127211031018] Helm RK Hans VP Reyna VF (2017) Trial by numbers. Cornell Journal of Law & Public Policy 27: 107.

[bibr25-13657127211031018] Helm RK Royer CE Ceci SJ (2018) Child interrogations and testimony. In: Koen W Bowers CM (eds) The Psychology and Sociology of Wrongful Convictions: Forensic Science Reform. London: Academic Press, pp.83–116.

[bibr26-13657127211031018] Hope L Gabbert F (2019) Memory at the sharp end: The costs of remembering with others in forensic contexts. Topics in Cognitive Science 11(4): 609–626. 10.1111/tops.12357 30073777

[bibr27-13657127211031018] Houston KA Hope L Memon A , et al. (2013) Expert testimony on eyewitness evidence: In search of common sense. Behavioral Sciences & the Law 31(5): 637–651. 10.1002/bsl.2080 24000168

[bibr28-13657127211031018] Howe ML Knott LM (2015) The fallibility of memory in judicial processes: Lessons from the past and their modern consequences. Memory 23(5): 633–656. 10.1080/09658211.2015.1010709 25706242 PMC4409058

[bibr29-13657127211031018] Innocent.org (2000) DNA Evidence eventually clears man in ‘shoe swap’ trial. https://innocentorguk.wordpress.com/2016/01/10/dna-evidence-eventually-clears-man-in-shoe-swap-trial-2000/

[bibr1-13657127211031018] *JH and TG*, case numbers 04/5576/D3 and 04/5577/D3.

[bibr30-13657127211031018] Jores T Colloff MF Kloft L , et al. (2019) A meta-analysis of the effects of acute alcohol intoxication on witness recall. Applied Cognitive Psychology 33(3): 334–343. 10.1002/acp.3533

[bibr31-13657127211031018] Judicial College (2020) Crown court compendium. https://www.judiciary.uk/wp-content/uploads/2020/12/Crown-Court-Compendium-Part-I-December-2020-amended-01.02.21.pdf

[bibr32-13657127211031018] Kahan DM Jenkins-Smith H Braman D (2011) Cultural cognition of scientific consensus. Journal of Risk Research 14(2): 147–174. 10.1080/13669877.2010.511246

[bibr33-13657127211031018] Kassin SM Sommers SR (1997) Inadmissible testimony, instructions to disregard, and the jury: Substantive versus procedural considerations. Personal and Social Psychology Bulletin 23(10): 1046–1054. 10.1177/01461672972310005

[bibr34-13657127211031018] Kassin SM Tubb VA Hosch HM , et al. (2001) On the’ general acceptance’ of eyewitness testimony research: A new survey of the experts. American Psychologist 56(5): 405–416. 10.1037/0003-066X.56.5.405 11355363

[bibr35-13657127211031018] Koehler JJ (2006) Train our jurors. In: Gigerenzer G Engel C (eds) Heuristics and the Law. Cambridge, MA: MIT Press, pp.11–21.

[bibr36-13657127211031018] Laney C Loftus EF (2010) Change blindness and eyewitness testimony. In: Davies GM Wright DB (eds) Current Issues in Applied Memory Research. New York: Psychology Press, pp.142–159.

[bibr37-13657127211031018] Lecci L Myers B (2008) Individual differences in attitudes relevant to juror decision making: Development and validation of the pretrial juror attitude questionnaire (PJAQ) 1. Journal of Applied Social Psychology 38(8): 2010–2038. 10.1111/j.1559-1816.2008.00378.x

[bibr38-13657127211031018] Leverick F (2020) What do we know about rape myths and juror decision making? The International Journal of Evidence & Proof 24(3): 255–279. 10.1177/1365712720923157

[bibr39-13657127211031018] Lieberman JD Krauss DA Heen M , et al. (2016) The good, the bad, and the ugly: Professional perceptions of jury decision-making research practices. Behavioral Sciences & the Law 34(4): 495–514. 10.1002/bsl.2246 27193481

[bibr40-13657127211031018] Loftus EF (2003) Make-believe memories. American Psychologist 58(11): 867–873. 10.1037/0003-066X.58.11.867 14609374

[bibr41-13657127211031018] Loftus EF Miller DG Burns HJ (1978) Semantic integration of verbal information into a visual memory. Journal of Experimental Psychology: Human Learning and Memory 4(1): 19–31. 10.1037/0278-7393.4.1.19 621467

[bibr42-13657127211031018] McAuliff BD Kovera MB Nunez G (2009) Can jurors recognize missing control groups, confounds, and experimenter bias in psychological science? Law and Human Behavior 33(3): 247–257. 10.1007/s10979-008-9133-0 18587635 PMC2860776

[bibr43-13657127211031018] National Registry of Exonerations (2021) Browse the national registry of exonerations. Law.umich.edu. Available at: https://www.law.umich.edu/special/exoneration/Pages/browse.aspx (accessed 13 April 2021).

[bibr44-13657127211031018] Papailiou A Yokum D Robertson C (2015) The novel New Jersey eyewitness instruction induces skepticism but Not sensitivity. PLOS ONE 10(12): e0142695. 10.1371/journal.pone.0142695 26650237 PMC4674112

[bibr45-13657127211031018] Pennington N Hastie R (1981) Juror decision-making models: The generalization gap. Psychological Bulletin 89(2): 246. 10.1037/0033-2909.89.2.246

[bibr46-13657127211031018] Pennington N Hastie R (1986) Evidence evaluation in complex decision making. Journal of Personality and Social Psychology 51(2): 242–258. 10.1037/0022-3514.51.2.242

[bibr47-13657127211031018] Pennington N Hastie R (1988) Explanation-based decision making: Effects of memory structure on judgment. Journal of Experimental Psychology: Learning, Memory, and Cognition 14(3): 521–533. 10.1037/0278-7393.14.3.521

[bibr48-13657127211031018] Pennington N Hastie R (1992) Explaining the evidence: Tests of the story model for juror decision making. Journal of Personality and Social Psychology 62(2): 189–206. 10.1037/0022-3514.62.2.189

[bibr49-13657127211031018] Penrod SD Cutler B (1999) Preventing mistaken convictions in eyewitness identification trials. In: Roesch R Hart SD Ogloff JRP (eds) Perspectives in Law & Psychology, Vol. 10. Psychology and Law: The State of the Discipline. Dordrecht: Kluwer Academic Publishers, pp.89–118.

[bibr50-13657127211031018] Reed AW (2007) The ABCs of tutoring your jury. TRIAL 43(10): 20–23.

[bibr51-13657127211031018] Reyna VF (2012) A new intuitionism: Meaning, memory, and development in fuzzy-trace theory. Judgment and Decision Making 7(3): 332–359. 25530822 PMC4268540

[bibr52-13657127211031018] Ross DF Marsil DF Benton TR , et al. (2006) Children’s susceptibility to misidentifying a familiar bystander from a lineup: When younger is better. Law and Human Behavior 30(3): 249–257. 10.1007/s10979-006-9034-z 16729204

[bibr53-13657127211031018] Ross DR Ceci SJ Dunning D , et al. (1994) Unconscious transference and mistaken identity: When a witness misidentifies a familiar but innocent person. Journal of Applied Psychology 79(6): 918–930. 10.1037/0021-9010.79.6.918

[bibr54-13657127211031018] Ruva C McEvoy C Bryant JB (2007) Effects of pre-trial publicity and jury deliberation on juror bias and source memory errors. Applied Cognitive Psychology 21(1): 45–67. 10.1002/acp.1254

[bibr55-13657127211031018] *R v Browning* (1995) Crim LR, 227.

[bibr56-13657127211031018] *R v Hallam* (2012) No. 2011/04293/C5.

[bibr57-13657127211031018] *R v H (JR) (Childhood Amnesia)* (2006) 1 Cr App R 195.

[bibr58-13657127211031018] *R v Kamara* (2000) EWCA Crim 37.

[bibr59-13657127211031018] *R v Nealon* (2014) EWCA Crim 574.

[bibr60-13657127211031018] *R v Pendleton* (2002) 1 WLR 72.

[bibr61-13657127211031018] *R v Turnbull* (1977) QB 224.

[bibr62-13657127211031018] *R v Turner* (1975) 1 All ER 70.

[bibr63-13657127211031018] Shaw JS III Garcia LA McClure KA (1999) A lay perspective on the accuracy of eyewitness testimony 1. Journal of Applied Social Psychology 29(1): 52–71. 10.1111/j.1559-1816.1999.tb01374.x

[bibr64-13657127211031018] Simons D Chabris C (2011) What people believe about how memory works: A representative survey of the U.S. Population. PLOS ONE 6(8): e22757. 10.1371/journal.pone.0022757 21826204 PMC3149610

[bibr65-13657127211031018] Simons DJ Chabris CF (2012) Common (mis)beliefs about memory: A replication and comparison of telephone and Mechanical Turk survey methods. PLOS one 7(12): e51876. 10.1371/journal.pone.0051876 23272183 PMC3525574

[bibr66-13657127211031018] Sklansky DA (2013) Evidentiary instructions and the jury as other. Stanford Law Review 65: 407– 456.

[bibr67-13657127211031018] Steblay NK Hosch HM Culhane SE , et al. (2006) The impact on juror verdicts of judicial instruction to disregard inadmissible evidence: A meta-analysis. Law and Human Behavior 30(4): 469–492. 10.1007/s10979-006-9039-7 16906469

[bibr68-13657127211031018] Thomas AK Loftus EF (2002) Creating bizarre false memories through imagination. Memory & Cognition 30(3): 423–431. 10.3758/BF03194942 12061762

[bibr69-13657127211031018] Thompson DM (1988) Context and false recognition. In: Davies GM Thompson DM (eds) Memory in Context: Context in Memory. Chichester: Wiley, 285–304.

[bibr70-13657127211031018] Wade K Garry M Don Read J , et al. (2002) A picture is worth a thousand lies: Using false photographs to create false childhood memories. Psychonomic Bulletin & Review 9(3): 597–603. 10.3758/bf03196318 12412902

[bibr71-13657127211031018] Wells GL Memon A Penrod SD (2006) Eyewitness evidence: Improving its probative value. Psychological Science in the Public Interest 7(2): 45–75. 10.1111/j.1529-1006.2006.00027.x 26158855

[bibr72-13657127211031018] Wise RA Safer MA (2004) What US judges know and believe about eyewitness testimony. Applied Cognitive Psychology: The Official Journal of the Society for Applied Research in Memory and Cognition 18(4): 427–443. 10.1002/acp.993

[bibr73-13657127211031018] Yaros JL Salama DA Delisle D , et al. (2019) A memory computational basis for the other-race effect. Scientific Reports 9(1): 1–11. 10.1038/s41598-019-55350-0 31853093 PMC6920375

